# Tissue culture-induced somaclonal variation of decreased pollen viability in torenia (*Torenia fournieri* Lind.)

**DOI:** 10.1186/1999-3110-54-36

**Published:** 2013-09-23

**Authors:** ShuLan Sun, JianQiang Zhong, ShuHua Li, XiaoJing Wang

**Affiliations:** Guangdong Key Lab of Biotechnology for Plant Development, College of Life Sciences, South China Normal University, Guangzhou, Guangdong, 510631 People’s Republic of China

**Keywords:** DNA methylation, Plant growth regulators, Pollen viability, Somaclonal variation, Torenia

## Abstract

**Background:**

Phenotypic and genotypic variations, collectively called somaclonal variations, are induced during tissue culture.

**Results:**

We studied the phenotypic variation in pollen viability of regenerants of torenia after subculturing for one to nine generations. We found that pollen viability of regenerants continuously decreased with increasing subculture time. High concentrations of plant growth regulators applied to the Murashige and Skoog (MS) medium also resulted in diminished pollen viability. Furthermore, antibiotic application during gene transformation also decreased pollen viability of the transformants. However, the process of long-term culture did not significantly change pollen viability. The mean methylation level of regenerants showed a 0.28% to 3.95% decrease in seedlings subcultured in vitro for nine generations. Moreover, when the ninth subcultured regenerants with reduced pollen vibility were recovered in soil to get seeds, the pollen viability of seed-derive plants was similar to that of the wild type.

**Conclusions:**

The results show that plant growth regulators, antibiotics, and the number of subculture generations influence somaclonal variations in torenia. The somaclonal variations in torenia may results from epigenetic changes.

**Electronic supplementary material:**

The online version of this article (doi:10.1186/1999-3110-54-36) contains supplementary material, which is available to authorized users.

## Background

Tissue culture is a powerful tool in plant gene transformation and molecular breeding. Phenotypic and genotypic variations, collectively called somaclonal variations, have long been known to be induced during dedifferentiation and regeneration of plants in tissue cultures (Larkin and Scowcroft 1981; Filipecki and Malepszy 2006). Several studies have reported the involvement of plant growth regulators used for regeneration of tissue culture and antibiotics used for transformant selection in somaclonal variations (LoSchiavo et al. 1989; Bregitzer et al. 1995; Schmitt et al. 1997; Rakoczy-Trojanowska 2002; Bardini et al. 2003). Factors such as explant genotypes and types of explants have also been known to play a role in somaclonal variations (Gaj 2004). Additionally, some studies show that accumulation of genetic changes during long-term culture *in vitro* also contribute to somaclonal variations (Peredo et al. 2006; Smýkml et al. 2007). Therefore, these data indicate the significance of determining the factors that influence the rate of variations during tissue culture and plant gene transformation.

The types of variations that are frequently observed may differ from species to species, and determining the nature of an observed variation is often complicated (Saunders et al. 1992). Genetic changes including point mutations, rearrangements in nuclear or organellar DNA, ploidy and activation of mobile elements were initially reported to be responsible for somaclonal variations (Jain 2001). However, a growing body of evidence has shown that epigenetic mutations, especially methylation variations, are involved (Kaeppler et al. 2000; Matthes et al. 2001; Xu et al. 2004; Peredo et al. 2006; Bednarek et al. 2007; Schellenbaum et al. 2008).

We recently observed that transgenic plants of torenia grown from explants subcultured for several generations are all male sterile. In this study, we investigated the factors that contribute to this type of somaclonal variation in torenia. Our results show that application of plant growth regulators and antibiotics, and the number of subculture generations influence somaclonal variations in torenia. Furthermore, we studied the methylation level of the regenerants from the ninth subcultured *in vitro* seedlings to investigate the effect of methylation level on somaclonal variations.

## Methods

### Plant materials and growth conditions

Seeds of torenia (*Torenia fournieri* Lind.) were surface-sterilized for 30 s in 70% EtOH and transferred to 10% NaClO for 10 min. The seeds were rinsed five times with sterile distilled water and germinated on half-strength Murashige and Skoog (MS) medium containing 1.5% sucrose solidified with 1% agar (basic MS medium; pH 5.8) at an ambient temperature of 24-26°C, a 16 h light (35 μmol m^-2^ s^-1^)/8 h dark photoperiod cycle and 65%-75% humidity. The germinated plants were labeled as R0 plants. For some experiments, the seeds were kept in a cold room (4°C) and sown directly into a well-watered potting mix.

### Sequential subculture of regenerated plants

For sequential subculture *in vitro*, the leaves of R0 shoots 5 cm in height grown in basic MS medium were cut into approximately 1 cm^2^ pieces. The leaf explants were plated on a shoot-regenerated medium (basic MS medium containing 1 mg/l 6-benzylaminopurine [6-BA] and 0.1 mg/l α-naphthalenehydrolysate [NAA]) for R1 shoot regeneration. The regenerated R1 shoots were then transferred to a basic MS medium for rooting and the R1 seedlings were used for further transplantation. The R1 leaf explants were used for R2 regeneration, and the regenerated R3–R9 shoots were obtained using the same procedure. The seedlings of R0–R9 with approximately 4 cm shoots were transplanted to a well-watered potting mix until flowering and seed setting.

### Pollen viability test

Methylthiazoletetrazolium (MTT) staining (Dafni and Firmage 2000) method was used for the pollen viability test. One hundred milligrams of MTT solution (Sigma) was dissolved in 5 ml of 5% sucrose solution and kept at 4°C. The sample was then mixed with one drop of MTT solution and air-dried. The pollen sample was mixed with another drop of MTT solution and observed under an Olympus BX51 microscope (Olympus, Japan). Dark purple pollens were recorded as viable, and those that were unstained were recorded as unviable. Two flowers per seedling were used, and 7 to 30 seedlings were examined for each test. More than 500 grains of pollen were assessed in each flower.

### Variation frequency of pollen viability

As all the plants obtained from seeds sown directly onto the soil showed greater than 80% pollen viability, the plants with pollen viability lower than 80% were identified as varied plants. The variation frequency of pollen viability is defined as the ratio of varied regenerants to total regenerants.

### Gene transformation

The vector plasmid pER10 used for gene transformation was obtained from Professor Nam-Hai Chua of Rockefeller University (New York, NY). The torenia plants were transformed with *Agrobacterium* harboring the empty vector pER10 as previously described (Aida and Shibata 1995), but with a different shoot regeneration medium as modified by Li et al. (2006). Transformants were selected based on kanamycin resistance and identified by PCR using *NPT II*-specific primers (5′-GGTGCCCTGAATGAACTGAC-3′ and 5′- TAGCCAACGCTATCTCCTGA- 3′).

### Extraction of genomic DNA and measurement of methylation level by high-performance capillary electrophoresis

Genomic DNA was extracted and purified using a MagExtractor kit (Toyobo, Japan) according to the manufacturer’s instructions. DNA samples (5 μg) were denatured in boiling water for 2 min and immediately transferred to ice. S1 buffer (1 μl) and S1 nuclease (0.8 μl) (Takara, Japan) were then added to the DNA samples. After incubating the mixtures overnight at 37°C, alkaline phosphatase (0.8 μl) (AP; Takara, Japan) and AP buffer (1.2 μl) were added and the mixture was incubated for an additional 2 h at 37°C. The samples were centrifuged for 20 min at 15,000 *g*, and the supernatant was analyzed in a capillary electrophoresis system (P/ACE™ MDQ, Beckman, USA). An uncoated fused silica capillary (570 × 0.075 mm^2^ i.d.; effective length, 500 mm; Polymicro Technologies) and a buffer containing 50 mM NaHCO_3_ and 70 mM SDS, pH 9.6, were used for capillary electrophoresis. The samples were injected at 0.5 psi for 5 s and the electrophoresis was run at 25°C at an operating voltage of 17 kV for 10 min. Absorbance was measured at 254 nm on-column. The relative methylation level of each DNA sample was quantified as the percentage of methyldeoxycytidine (mdC) to total deoxycytidine (dC + mdC), as described by Smýkml et al. (2007).

## Results

### Significantly decreased pollen viability of regenerated plants resulting from sequential subculture

**Figure 1:**
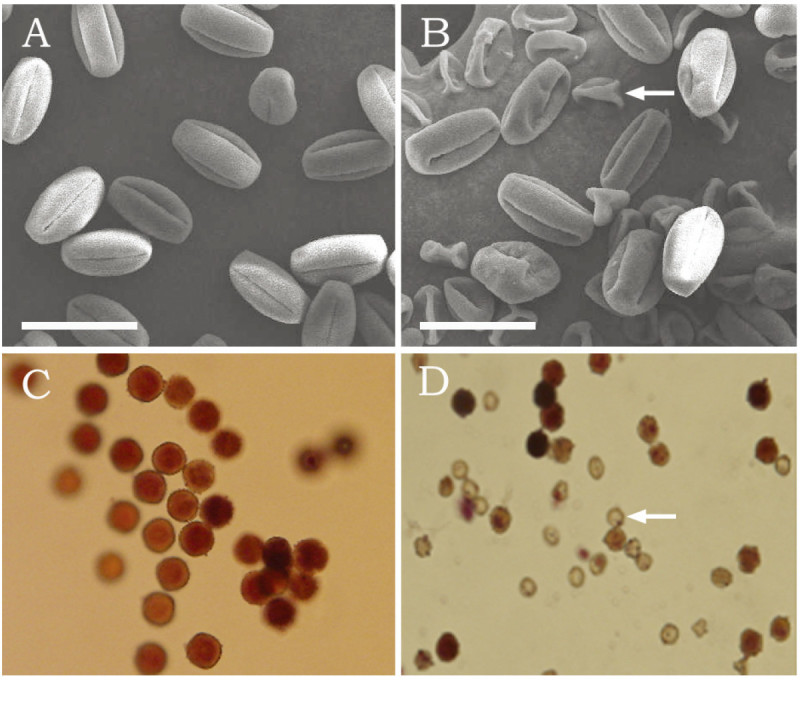
**Pollen abortion in regenerants detected by scanning electron microscopy (SEM) and MTT staining. (A)** SEM image of mature pollen of plants derived from seeds directly sown into the soil; **(B)** SEM image of mature pollen from regenerants with high pollen abortion rate. The SEM images (**A** and **B**) have the same magnification. The bar represents 50 μm, and the arrow indicates aborted pollen; **(C)** MTT staining of mature pollen of plants derived from seeds sown directly into the soil; **(D)** MTT staining of mature pollen of regenerants with high pollen abortion rate. The arrow indicates aborted pollen.

**Figure 2:**
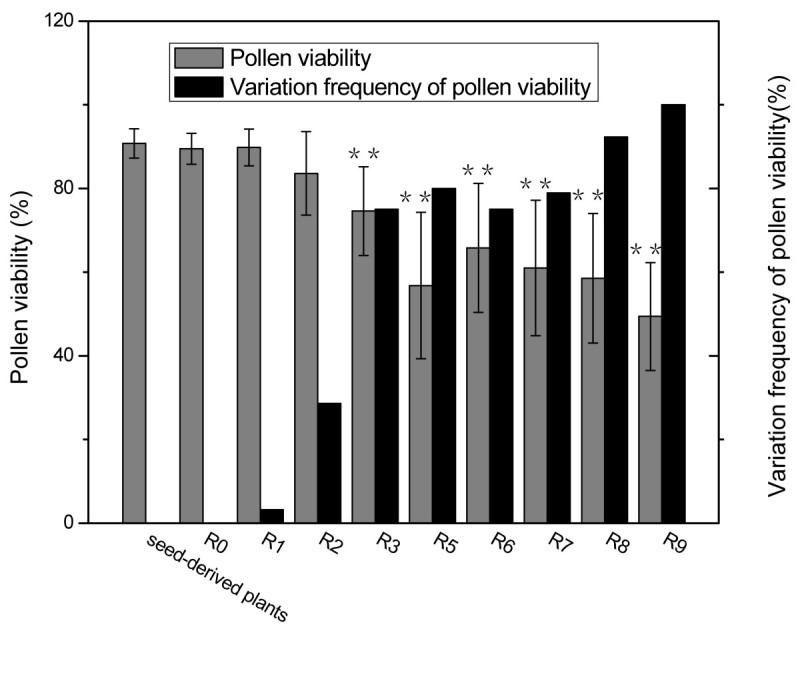
**Decreased pollen viability and increased variation frequency of pollen viability in regenerated plants of torenia.** ^**^p < 0.05, compared with R0.

Pollen abortion occurred in a few regenerated plants obtained from tissue cultures (Figure 1). The pollen viability of each generation of regenerated plants was examined using MTT staining. As shown in Figure 2 and Table 1, pollen viability did not differ between plants directly germinated and those grown in soil and R0. Although the mean pollen viability of R1 plants did not decrease significantly compared with R0, one R1 plant showed significantly decreased pollen viability. The difference between the variation frequency of pollen viability in R0 and that in R1 was evident. The mean pollen viability of R2 did not decrease significantly, but the variation frequency of its pollen viability increased to 28.6% compared with 0 in R0 and 3.2% in R1. The mean pollen viability decreased significantly and the variation frequency of pollen viability increased to 75% until R3. The mean pollen viability of the regenerants decreased gradually with increase in sequential subculture time, except for the 56.8% pollen viability of R5, which is lower than the values for R6 and R7. This may be attributed to the small number of samples assessed in R5. The mean pollen viability of R9 plants decreased to 49.4%, but the variation frequency of its pollen viability increased up to 100%. When self-pollinated, the R3–R9 plants still set seeds (data not shown), indicating that those plants were not completely 'male sterile’.

**Table 1:** **Pollen viability of torenia regenerants in each subculture generation**

Genotype	Seed-derived plants		R0	R1		R2	R3	R5	R6	R7		R8	R9
Pollen viability of individual detected plant (%)	81.4	84.8	82.2	74.3	81.1	64.4	62.0	34.7	49.4	42.1	44.7	37.8	24.3
	85.1	87.2	85.6	84.5	85.5	77.2	66.7	42.5	52.4	44.9	45.8	45.1	33.0
	88.6	88.9	86.7	85.9	86.1	84.3	86.5	44.2	52.5	47.3	48.2	46.2	46.3
	89.5	90.3	89.5	87.1	88.1	85.6	71.2	47.8	53.4	48.9	52.0	48.6	47.0
	90.5	90.6	89.5	88.1	88.8	90.1		51.3	53.7	55.1	56.8	49.2	51.1
	90.7	91.5	90.8	89.1	89.9	90.5		55.2	54.9	57.0	61.0	52.1	51.8
	91.9	91.9	91.4	90.3	90.6	93.0		55.9	55.3	62.8	66.3	52.2	54.4
	92.5	92.7	92.6	90.7	91.1			63.7	55.9		78.8	54.8	56.2
	93.2	93.3	93.0	91.2	91.7			85.4	60.6		85.8	55.3	62.1
	93.5	93.8	94.1	91.9	91.9			87.2	66.9		86.4	77.3	67.8
	94.3	95.7		92.1	92.1				67.3		87.0	77.5	
		96.3		92.2	92.3				75.5		87.4	77.7	
				92.4	92.8				84.0			86.2	
				93.5	94.0				84.4				
				94.5	94.7				90.8				
					95.3				95.1				

### Pollen viability of regenerated plants decreased with increasing growth regulator concentration

Different concentrations of 6-BA and NAA were used to induce regeneration of shoots from R0 explants and to test the effects of plant regulators on variation in pollen viability. When the concentration of 6-BA was increased to 5 mg/l, which was five times that in the primary shoot induction medium, while keeping the concentration of NAA the same as that in the primary medium (0.1 mg/l), the mean pollen viability of the R1 plants decreased slightly. However, one of seven regenerated R1 plants showed remarkably decreased pollen viability. Similar results were obtained when only the concentration of NAA was increased to 0.5 mg/l in the shoot induction medium. Under this condition, the mean pollen viability of the R1 plants decreased slightly and one of eight regenerated R1 plants showed remarkably decreased pollen viability. However, when the concentrations of both 6-BA and NAA were increased to five times those in the primary shoot induction medium (5 mg/l 6-BA and 0.5 mg/l NAA), the mean pollen viability was significantly lower (78.9%) than in the control (88.7%) and the variation frequency of pollen viability increased to 42.9% (Table 2).

**Table 2:** **Pollen viability and variation frequencies of pollen viability in R1 plants induced by different concentrations of plant growth regulators**

Growth regulator (mg l^-1^)		Pollen viability (%)	Number of detected plants	Variation frequency of pollen viability
6-BA	NAA			
0	0	88.7 ± 2.6a	3	0
1	0.1	89.8 ± 4.4a	31	3.2
5	0.1	85.8 ± 5.9a	7	14.3
1	0.5	85.2 ± 11.7a	8	12.5
5	0.5	78.9 ± 9.7b	7	42.9

Data are expressed as mean ± SD. Values followed by different letters differ significantly (DPS v3.01). *p* < 0.05.

### Decreased pollen viability of transgenic plants with antibiotic addition

Torenia with the pER10 empty vector was transformed using R0 leaf explants to determine other factors that may induce somaclonal variations during gene transformation. The kanamycin-resistant regenerated shoots were transferred into soil and subsequently verified by PCR (data not shown). The mean pollen viability of the transgenic plants significantly decreased (53.5%) compared with that of the untransformed control (88.3%), whereas the variation frequency of pollen viability increased to 75% (Table 3). Since R1 and the transgenic plants derived from R0 leaf explants differed only in the addition of kanamycin, these results indicate that the antibiotic affected somaclonal variation in torenia.

**Table 3:** **Decreased pollen viability in transgenic plants of torenia selected by kanamycin resistance**

Genotype	Pollen viability (%)	Number of detected plants	Variation frequency of pollen viability
R0	88.3 ± 2.7a	3	0
Transgenic plants	53.5 ± 24.3b	8	75.0

Data are expressed as mean ± SD. Values followed by different letters differ significantly (DPS v3.01). *p* < 0.05.

### Variation of decreased pollen viability could not be inherited in the sexual generation

Genetic and epigenetic variations have been reported to be heritable and non-heritable, respectively (Jain 2001). The R5 plants with high rates of pollen abortion were pollinated with pollen from wild type plants to investigate the cause of this phenomenon in regenerated plants. We observed that three progenies of pollinated plants recovered pollen viability (Table 4). Thus, variations in decreased pollen viability in the R5 plants could not be inherited in the sexual generation, suggesting that this variation is epigenetic.

**Table 4:** **Pollen viabilities of progenies of an R5 plant with high pollen abortion rate crossed with plants derived from seeds directly sown into the soil**

Genotype	Pollen viability (%)	Pollen viability of self-pollination-derived progeny lines (%)
R5	42.5	91.4
		92.5
		95.2

### Decrease in the global DNA methylation levels of R9 plants

The non-inherited variation of decreased pollen viability in seed-derived generations indicates the possibility of an epigenetic variation in regenerated plants. The global DNA methylation levels of the R9 regenerated plants with varying degrees of decreased pollen viability were further detected. The mean methylation level of the R0 plants was 12.78%, whereas that of the R9 plants decreased by 0.28%–3.95% compared with that of the R0 plants (Table 5).

**Table 5:** **Global DNA methylation levels and pollen viabilities of R0 and R9 plants**

Regenerants	% mdC	Pollen viability (%)
R0#1	13.85 ± 0.35	91.9
R0#2	13.39 ± 0.51	84.8
R0#3	11.11 ± 0.75	90.3
R9#1	12.50 ± 0.27	62.1
R9#2	11.63 ± 0.42	24.3
R9#3	9.90 ± 0.38	46.3
R9#4	8.83 ± 0.25	47.0

All experiments were repeated three times.

## Discussion

Most transformation protocols currently available for plants require tissue culture and the use of selectable markers, which exert stress that may result in somaclonal variations. Four factors that could affect the frequency of somaclonal variations were determined in this study. The pollen viability of plants regenerated from sequentially subcultured explants was examined. Pollen viability gradually decreased from R3 to R9 and was significantly reduced at R9 compared to that at R0. The variation frequency of pollen viability continuously increased from R1 to R9. Prolonged culturing has previously been reported to contribute to accumulation of genetic changes (Peredo et al. 2006). However, the leaves of late regenerants have been shown to exhibit significantly less genetic and epigenetic divergence from the source leaves than those exposed to short periods of callus growth (Rodríguez López et al. 2010). When torenia explants were subcultured on the same shoot induction medium for 20, 80, and 120 days, the regenerated R1 plants showed similar pollen viability as the control (data not shown). This suggests that prolonged explant culturing is not associated with the accumulation of somaclonal variations in torenia.

Plant growth regulators have previously been reported to influence the degree of somaclonal variations and to induce an increase in DNA methylation (LoSchiavo et al. 1989; Bregitzer et al. 1995). In the current study, when the concentrations of 6-BA and NAA were both increased to five times those in the primary shoot induction medium, the mean pollen viability was significantly lower (78.9%) than that of the control (88.7%) and the variation frequency of pollen viability also increased to 42.9%. This indicates that both growth regulators contributed to the accumulation of somaclonal variations. Aside from plant growth regulators, antibiotic application is also known to be associated with tissue culture-induced variations. In particular, Kanamycin has been reported to cause hypermethylation in tobacco plants (Schmitt et al. 1997; Rakoczy-Trojanowska 2002; Bardini et al. 2003). Kanamycin was hence used as a selection agent to generate transgenic plants in our study. The mean pollen viability of the transgenic plants grown in the presence of antibiotics was 53.5%, which is significantly lower compared to that of the control (88.3%). Additionally, the variation frequency of pollen viability also increased to 75% in the transgenic plants. Taken together, our results show that plant growth regulators, antibiotics, and the number, not the duration, of subculture generations influence somaclonal variations in torenia.

An intriguing observation made in our study is that the regenerated plants showed an almost uniform phenotype variation (i.e., decreased pollen viability) regardless of the stress exerted, suggesting that there are pre-existing mutations in the explants. However, the sequential accumulation of mutations with increasing subculture time indicates that mutations also occur during the culture process as previously reported (Kaeppler et al. 2000).

Kuznetsova et al. (2005) investigated decreased pollen viability in pea regenerants and detected completely sterile pollen in pea plants regenerated from long-term callus cultures. They did not find relation between genetic variations and duration on tissue culture medium, but they did not examine epigenetic variations of the regenerants (Kuznetsova et al. 2005). In the current work, we found that the transgenic plants with the expression vector incorporated with nine genes were all infertile (data not shown), indicating that the variation of infertility in all the transgenic lines could not have resulted from the insertion effect or the function of inserted genes (Filipecki and Malepszy 2006). Furthermore, we found that the variation of decreased pollen viability in seed-derived generations is not inherited, indicating that the variation in the regenerated plants is epigenetic. Many studies have shown that changes in the level and pattern of DNA methylation are involved in somaclonal variations, and changes in methylation have been detected in almost every study on somaclonal variations (Guo et al. 2007; Li et al. 2007). We found that the methylation level of the R9 plants decreased 0.28% to 3.95% compared with that of the R0 plants. Frequent reversions to the normal phenotype of somatic embryo-derived oil palm were also shown to be associated with a 2%-4% decrease in methylation (Rival et al. 1998; Jaligot et al. 2000; Matthes et al. 2001). Although Jaligot et al. (2004) found poor correlation between somaclonal variations and the methylation pattern using methylation-sensitive amplified polymorphism (MSAP), this method can still reveal changes in methylation levels in small regions that are masked in global measurements (Smýkml et al. 2007). In the case of peas, no significant difference was found at the DNA methylation level, but subtle genomic DNA mutations/rearrangements were detected only by amplified fragment length polymorphism (Smýkml et al., 2007).

Somaclonal variation has the potential of generating valuable variants in plant breeding. However, it may be an unintended or even undesired issue when the main objective is simply gene transformation. Having a more precise understanding of the stress factors that result in genetic and epigenetic changes and the mechanisms by which they contribute to somaclonal variations would be very useful in reducing the extent of variations (Boitel-Conti et al. 2000). Although diagnosing tissue culture-induced somaclonal variations is impossible, circumventing the variations using explants from seed-derived plants and with the concentration of growth regulators kept as low as possible during the course of transformation is recommended. Selection markers, such as green fluorescent protein (GFP), should ideally be chosen over antibiotics.

## Conclusions

In this paper, the factors that could affect the frequency of somaclonal variations were determined. The results show that plant growth regulators, antibiotics, and the number, not the duration, of subculture generations influence somaclonal variations in torenia. However, the nature of an observed variation is often complicated. We found that the mean methylation level of regenerants showed a 0.28% to 3.95% decrease in seedlings subcultured *in vitro* for nine generations, which indicate that the epigenetic nature of somaclonal variations exsit in torenia.

## Authors’ original submitted files for images

Below are the links to the authors’ original submitted files for images. Authors’ original file for figure 1 Authors’ original file for figure 2

## Abbreviations

6-BA: 6-benzylaminopurine
AP: Alkaline phosphatase
mdC: Methyldeoxycytidine
MSAP: Methylation-sensitive amplified polymorphism
NAA: α-naphthalenehydrolysate
MTT: Methylthiazoletetrazolium
SEM: Scanning electron microscopy.
