# Supplementary figures and images for: Tissue culture-induced somaclonal variation of decreased pollen viability in torenia (Torenia fournieri Lind.)
Source: Bot Stud. 2013 Sep 23;54:36. doi: 10.1186/1999-3110-54-36 (PMC5432822; doi:10.1186/1999-3110-54-36)

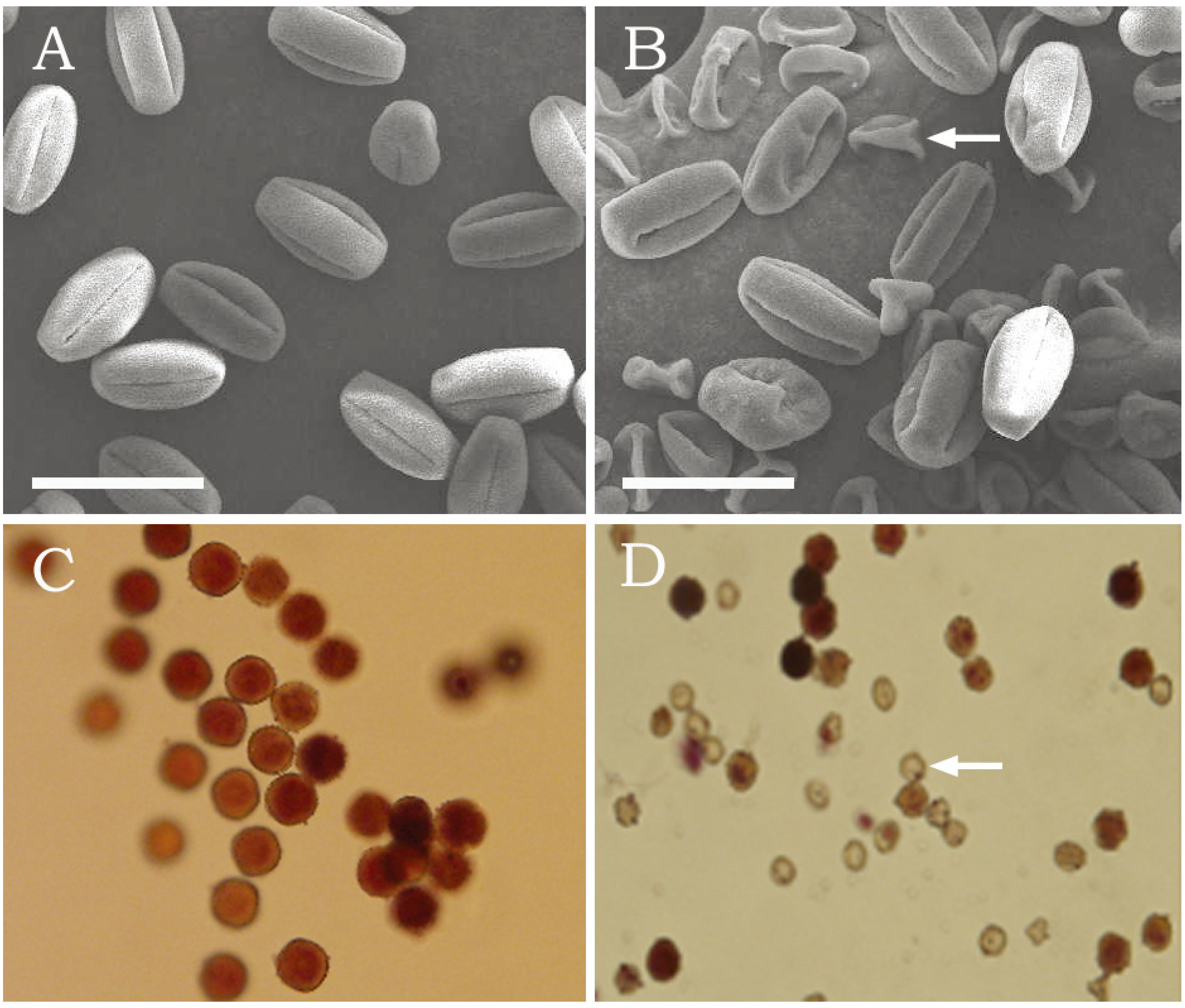

Supplement: Supplementary file 1 — Authors’ original file for figure 1 [file 40529_2013_40_MOESM1_ESM.tiff]

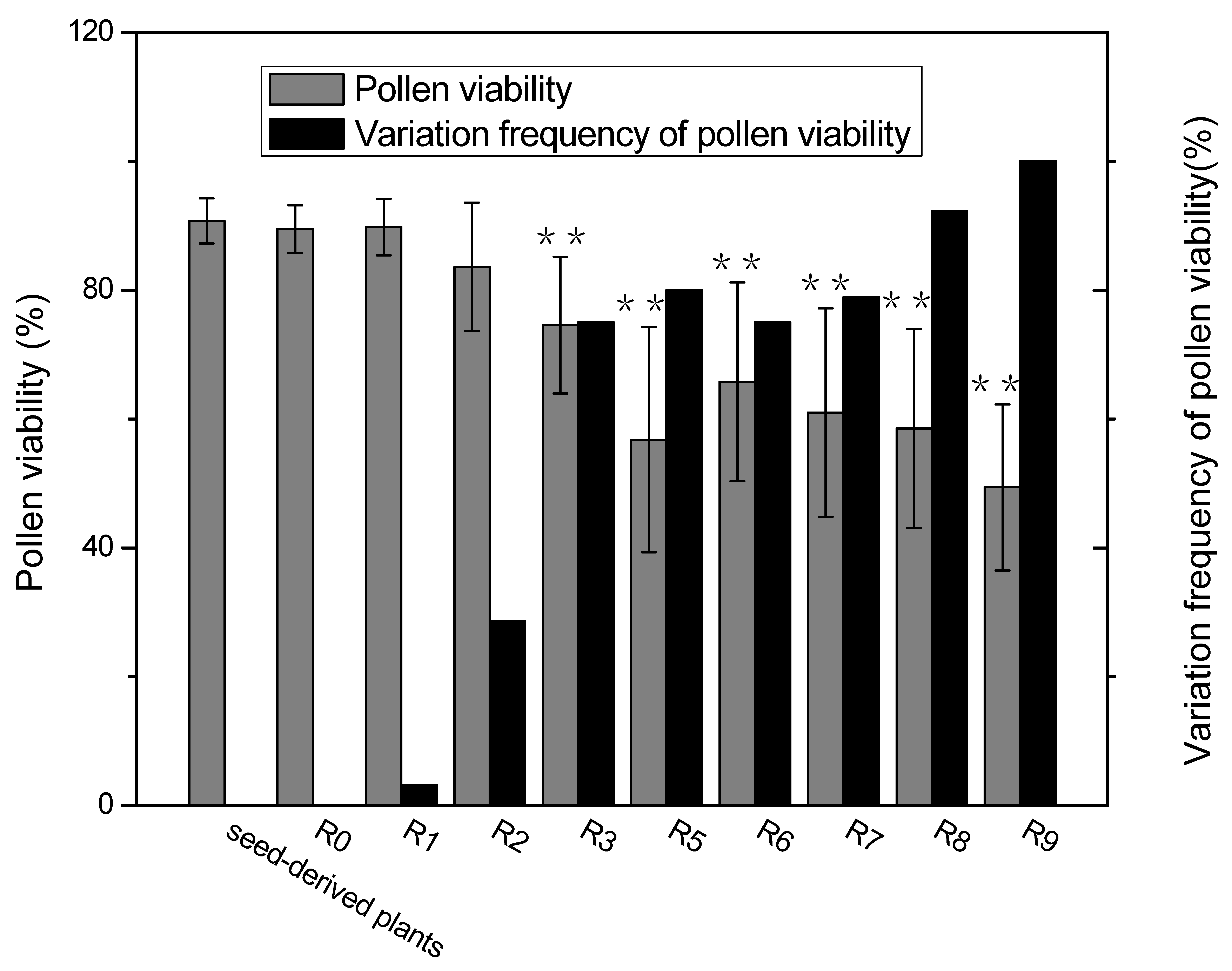

Supplement: Supplementary file 2 — Authors’ original file for figure 2 [file 40529_2013_40_MOESM2_ESM.tiff]
